# Acoustic Measures Capture Speech Dysfunction in Spinocerebellar Ataxia

**DOI:** 10.1002/acn3.70264

**Published:** 2025-11-28

**Authors:** Zena Fadel, Charlotte Hennessey, Hannah Lee, Pia Parekh, Sheng‐Han Kuo, Ami Kumar

**Affiliations:** ^1^ Initiative for Columbia Ataxia and Tremor Columbia University New York New York USA; ^2^ Teachers College Columbia University New York New York USA; ^3^ University of Rochester Rochester New York USA; ^4^ Department of Neurology, College of Physicians and Surgeons Columbia University New York New York USA

**Keywords:** AVQI, connected speech, formant analysis, spinocerebellar ataxia, sustained vowel

## Abstract

**Objective:**

Spinocerebellar ataxias (SCA) are hereditary cerebellar degenerative disorders with a common feature of dysarthria, involving impaired phonatory and articulatory control of speech, thereby affecting social communication. In this study, we investigated whether acoustic measures could objectively measure speech dysfunction and identify common indicators across SCA types 1, 2, and 3.

**Methods:**

We recorded speech from 24 SCA patients and 24 age‐matched controls during connected speech and sustained vowel tasks. Speech was assessed using the Acoustic Voice Quality Index (AVQI) and sub‐metrics (cepstral peak prominence smoothed [CPPS], harmonics‐to‐noise ratio [HNR], and shimmer). Jitter was used to quantify dysfunction in sustained vowels, and formant analysis was used to examine articulatory control. We also evaluated whether these measures capture speech dysfunction severity.

**Results:**

AVQI was the most consistent indicator of speech dysfunction across SCA subgroups, showing a significant increase compared to controls. SCA patients exhibited abnormalities in AVQI sub‐metrics (CPPS, HNR, and shimmer) and increased jitter during sustained vowels. Furthermore, formant analysis revealed articulation differences linked to changes in F2, F3, and F4 during connected speech, and F2 and F4 during sustained vowels. Additionally, AVQI correlates with the Scale for Assessment and Rating of Ataxia (SARA) speech sub‐scores, whereas CPPS and jitter can differentiate between mild and moderate stages of ataxic speech.

**Conclusion:**

Our findings show that SCA patients exhibit speech dysfunction across both connected speech and sustained vowel tasks, as AVQI stands out as a key indicator, whereas CPPS and jitter track ataxic speech severity. Our findings support using acoustic measures for objectively assessing speech dysfunction and thereby informing disease monitoring and guiding future targeted therapies.

## 1 Introduction

Spinocerebellar ataxias (SCAs) are a group of genetically inherited, progressive cerebellar degenerative disorders characterized by motor incoordination [1, 2]. Among the various phenotypic manifestations of SCA, speech disturbances, collectively referred to as ataxic dysarthria, are highly prevalent and significantly impact quality of life. The key brain area involved in SCAs is the cerebellum and its connected brain regions such as the brainstem. Given that the cerebellum plays a critical role in the coordination and timing of speech production, including regulation of respiration, phonation, articulation, and prosody [3], SCA patients thus have dysarthria characterized by slow, slurred speech with irregular articulation, altered voice quality, and variations in strength, speed, and tone. These impairments make verbal communication difficult and disrupt phonatory and articulatory control, which in turn impairs social interaction [4, 5]. While these symptoms are routinely noted during clinical assessments, we currently lack a detailed characterization of speech deficits in SCA that can serve as objective measures to monitor disease severity to guide therapeutic development. In addition, the existing rating scales to measure speech dysfunction in SCA lack granularity to capture subtle changes; therefore, objective acoustic measures are essential to complement clinical evaluations.

Recent advances in digital acoustic speech analysis offer objective, sensitive, and reproducible measures that are well suited for quantifying speech dysfunction across neurological disorders [6, 7]. Among these tools are the Acoustic Voice Quality Index (AVQI) [8, 9] and its sub‐metrics, Cepstral Peak Prominence‐Smoothed (CPPS) [10], harmonics‐to‐noise ratio (HNR) [11], shimmer, as well as measures such as jitter and formant frequencies [12, 13, 14]. These acoustic parameters capture subtle changes in voice quality, including breathiness, irregular vocal fold vibration, pitch and amplitude instability, and deviations in articulatory precision. Several studies have shown the potential of these measures in tracking disease severity in disorders such as amyotrophic lateral sclerosis and Parkinson's disease [15, 16, 17, 18, 19]. In amyotrophic lateral sclerosis, jitter and articulatory‐acoustic analyses were used to study tongue movements and dysarthria [15], and multiple acoustic measures, including jitter, shimmer, and HNR, were used to assess dysphonia [16]. In Parkinson's disease, jitter, shimmer, and other acoustic measures were used to characterize speech changes and the effects of levodopa therapy [17], while the AVQI has been applied both in cross‐sectional assessments [18] and to evaluate therapy‐related changes [19].

Importantly AVQI integrates multiple acoustic parameters into a single validated composite score, providing an interpretable measure of overall speech impairment. Unlike individual acoustic features that capture isolated aspects of phonation or articulation, AVQI simultaneously assesses sustained vowels and connected speech, offering a more comprehensive and clinically comparable index. Moreover, the quantitative nature of AVQI makes it particularly suitable for longitudinal monitoring and treatment response evaluation. However, prior studies using acoustic measures in cerebellar ataxia have not identified if AVQI can be the single metric that reliably captures speech impairment severity across multiple SCA subtypes, as they have typically focused on individual subtypes [20, 21, 22, 23, 24]. Even though SCA1, SCA2, and SCA3 differ in atrophy patterns and progression rates, they share core deficits in cerebellar timing and coordination that affect speech. These three subtypes are among the most prevalent SCAs and are well‐characterized in terms of genetic etiology and clinical progression, making them ideal for cross‐subtype comparisons.

Therefore, in this study we aim to identify a common acoustic marker that reflects shared dysfunction, providing a unified, objective measure of speech impairment suitable for cross‐subtype comparisons and informing disease monitoring and the usage of acoustic measures in future targeted interventions and disease‐modifying trials.

## 2 Methods

### 2.1 Patients

In this study, we recruited SCA1 (*n* = 6), SCA2 (*n* = 8), and SCA3 (*n* = 10) patients from the Ataxia Center of Excellence at Columbia University Irving Medical Center. We recruited age‐matched controls (*n* = 24) without a history of neurological disorders. The inclusion criteria for the SCA patients were, (1) confirmed SCA diagnoses by genetic tests identifying pathological repeat expansions of *ATXN1* [25], *ATXN2* [26], and *ATXN3* [27], and the presence of symptoms of cerebellar ataxia, (2) age 18–75, (3) with no history of traumatic brain injury or other neurological diagnosis that could interfere with speech function, and (4) could speak, read and understand English fluently. The speech sub‐scores of the Scale for Assessment and Rating of Ataxia (SARA) [28], a clinical tool, were also used to assess the severity of speech dysfunction in SCA patients. The uniform study protocol was approved by the Institutional Review Board of Columbia University, and informed consent was obtained from all participants.

### 2.2 Speech Recordings

Speech was recorded using a condenser microphone (Logitech Blue Snowball) and analyzed using the Praat software (version 6.4.12) [29]. Praat is validated software extensively used for assessing speech [6, 30, 31, 32]. The sampling frequency of all the recordings was 44,100 Hz and all files were saved in the .*wav* format. Two speech tasks were recorded, consisting of connected speech and a sustained vowel task. For the connected speech task, all patients and controls were asked to read a passage from “The Caterpillar,” [33] a phonemically balanced text often used in speech studies. The paragraph incorporates diverse speech functions and simple syntax, designed to highlight speech production while minimizing cognitive load for participants [31, 34]. For the sustained vowel task, all patients and controls completed two trials of producing the /a/ sound for as long as possible, with a required minimum duration of 3 s per trial.

### 2.3 Acoustic Measures

#### 2.3.1 AVQI and Sub‐Metrics

To quantify speech dysfunction across tasks, we assessed voice quality in both sustained vowel and connected speech recordings using AVQI, with a Praat script (AVQI v.03.01) [6]. AVQI compares a subject's connected speech and sustained vowel recordings to measure six acoustic markers (CPPS, HNR, shimmer local percentage, shimmer local dB, general slope of the long‐term average spectrum, and tilt of the regression line through the long‐term average spectrum), yielding an overall rating of voice quality [6, 8]. For the AVQI analysis, we extracted (1) 3 s of the sustained vowel task, and (2) a connected speech sentence (“My most MEMORABLE moment was riding on the Caterpillar.”) from the reading passage. This particular sentence, located later in the passage, was chosen because patients were more likely to have settled into a natural speech rhythm by that point. Importantly, all patients, including those with more severe impairments, were consistently able to speak this sentence, making it suitable for reliable comparison across participants. After the extraction of the connected speech and sustained vowel, the time was reset to 0 for standardization. Following this, a power cepstrogram was constructed for both files (settings involved: 60 Hz for pitch floor, 0.002 for time step(s), 5000 Hz for maximum frequency, and 50 Hz for pre‐emphasis) and the CPPS, an acoustic measure that quantifies the strength and regularity of the vocal signal's periodic structure was extracted using the following parameters: time averaging window of 0.01, a quefrency averaging window of 0.01, a trend line quefrency ranging from 0.001 to 0, a straight trend type and a robust fit method. The AVQI v.03.01 Praat script [6] was then run to produce an AVQI image with relevant metrics. Key sub‐metrics of interest extracted from this output included CPPS [10], HNR (indicating the relative strength of the harmonic component versus the noise component in the voice) [11], and shimmer (reflecting amplitude perturbations) [12, 14].

#### 2.3.2 Jitter

Given that sustained vowel performance is often under‐assessed in clinical settings, we applied acoustic metrics to better characterize phonatory dysfunction in this task, with phonatory control referring to the stability and quality of voice production. Specifically, we calculated jitter [12, 14], a measure of frequency perturbation that is particularly informative in sustained vowels, as it captures subtle microvariations in pitch stability indicative of impaired phonatory control. The corresponding audio (.*wav*) files were uploaded into Praat and analyzed using the voice report function. For comparison, jitter was also calculated for connected speech recordings.

#### 2.3.3 Formant Analysis

To better understand articulatory dysfunction, which refers to the control and coordination of the tongue, lips, jaw, and other vocal tract structures to produce speech sounds accurately, we performed formant analysis on both connected speech and sustained vowel recordings from all patients and controls. Formant analysis examines the acoustic properties of speech by measuring resonant frequencies known as formants produced by the vocal tract during speech production [13, 34, 35]. For connected speech, we used the same sentence and the sustained /a/ vowel trial extracted for AVQI analysis, applying formant tracking to these recordings to enable precise formant measurement and consistent comparisons between patients and controls while minimizing variability in speech performance. We extracted the first four formants (F1–F4) from each recording, capturing values at every time point and averaging them to obtain representative measures. F1 is primarily associated with tongue height, F2 with tongue advancement, F3 with lip and jaw positioning, and F4 is thought to reflect finer articulatory and vocal tract resonance characteristics [36, 37, 38, 39, 40]. This analysis offers insight into the structure and function of articulatory control in speech.

### 2.4 Stratified Analysis by Speech Dysfunction Severity

To examine whether acoustic metrics capture stage‐specific changes for distinguishing early from advanced speech impairment, we stratified SCA patients into two groups based on their SARA speech sub‐scores: (1) mild (scores 0–2) and (2) moderate‐to‐severe (scores 3–6). Acoustic measures, including AVQI, CPPS, HNR, shimmer, jitter, and formant frequencies were compared across controls, mild SCA, and moderate‐to‐severe SCA groups.

### 2.5 Statistical Analysis

Group comparisons of AVQI scores and individual sub‐metric acoustic measures were analyzed using independent *t*‐tests. Data distributions were assessed for normality and when normality assumptions were violated, non‐parametric Mann–Whitney *U* tests were applied for pairwise comparisons. Multiple comparisons were corrected using the Holm–Šidák method with a significance threshold set at *p* < 0.05. For comparisons across speech SARA severity subgroups, one‐way ANOVA was used when assumptions were met, followed by Tukey's post hoc test. For comparisons across more than two groups where normality was not met, the Kruskal–Wallis test was performed, followed by Dunn's multiple comparisons test. The relationship between the duration of connected speech and SARA speech sub‐scores was assessed using Spearman's correlation due to non‐normal data distribution, whereas Pearson correlation was used to evaluate the association between AVQI and SARA speech sub‐scores.

## 3 Results

### 3.1 Patient Demographics

A total of 48 participants were recruited for this study, including 24 healthy controls and 24 SCA patients. The patient group involved 6 SCA1, 8 SCA2, and 10 SCA3 patients. Groups were age‐matched, with the control group averaging 45.75 ± 15.49 years old and the SCA group averaging 46.88 ± 12.32 years old. The average age of disease onset for the SCA group was 33.51 ± 14.30 years, with an average disease duration of 11.50 ± 5.63 years. SCA patients had a total SARA score of 18.44 ± 6.19 and a SARA speech sub‐score of 2.25 ± 1.22. Table 1 summarizes the participant demographic characteristics.

**TABLE 1:** Demographic data of subjects.

	Controls	All SCA (1, 2, 3)	SCA 1	SCA 2	SCA 3
*n*	24	24	6	8	10
Age (years)	45.75 ± 15.49	46.88 ± 12.32	48.67 ± 12.64	44.88 ± 10.72	47.40 ± 14.28
Age of onset (years)	N/A	33.51 ± 14.30	31.51 ± 19.74	32.25 ± 12.03	35.70 ± 13.09
Disease duration (years)	N/A	11.50 ± 5.63	9.68 ± 6.54	12.63 ± 5.18	11.7 ± 5.66
*Gender*					
Male	58.33%	33.33%	50%	37.50%	30%
Female	41.66%	66.66%	50%	62.50%	70%
Total SARA	N/A	18.44 ± 6.19	16.5 ± 4.84	19.38 ± 5.88	18.85 ± 7.38
SARA speech	N/A	2.25 ± 1.22	2.67 ± 1.03	2.38 ± 0.92	1.90 ± 1.52

*Note*: Values represent mean ± standard deviation.

Abbreviations: N/A, not applicable; SARA, Scale of Assessment and Rating of Ataxia.

### 3.2 Connected Speech Duration

We first examined the duration for the connected speech sentence (“My most MEMORABLE moment was riding on the Caterpillar.”) and found that SCA patients spent 63.79% more time completing the sentence as compared to healthy controls (mean of 5.70 ± 2.60 s vs. 3.48 ± 1.36 s; *p* < 0.0001, Figure S1A,B) indicating reduced speed of the speech. We further correlated the duration of SCA speech with SARA speech sub‐scores and found a significant positive correlation (*r* = 0.5751, *p* = 0.0033; Figure S1C), indicating that longer speech duration is associated with greater speech impairment severity in SCA patients.

### 3.3 AVQI

**FIGURE 1:**
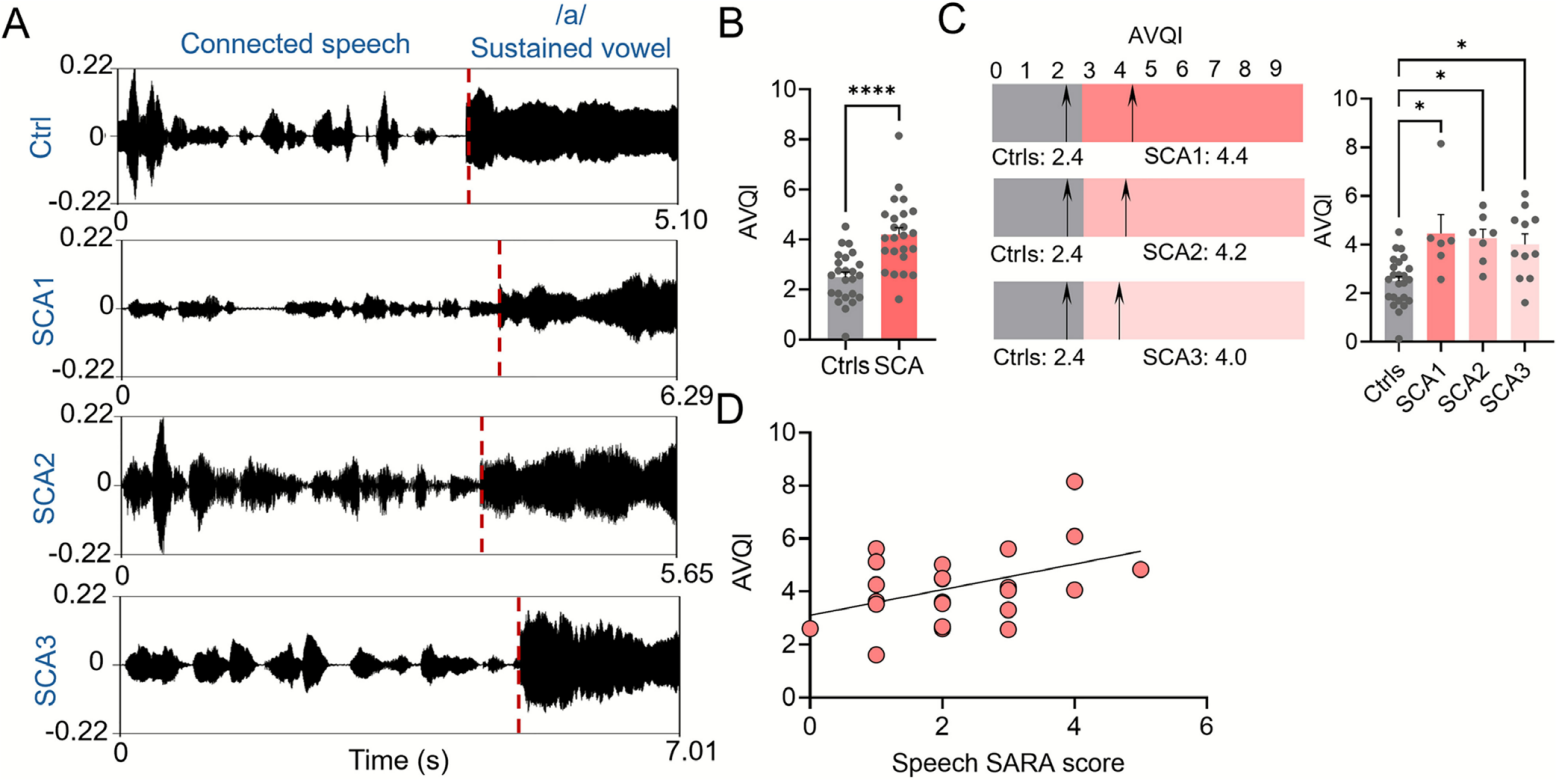
Acoustic voice quality index (AVQI) analysis. (A) Representative waveforms from connected speech and sustained vowel (/a/) recordings in a control subject and patients with SCA1, SCA2, and SCA3. Red dashed lines indicate transitions between tasks. (B) Group comparison of AVQI scores between controls and all SCA patients, showing significantly elevated values in the SCA group (****p < 0.0001). (C) AVQI comparisons between controls and individual SCA subtypes. Arrows indicates group means. All SCA subtypes show significantly higher AVQI scores compared to controls (*p < 0.05). (D) Positive correlation between AVQI and speech‐specific Scale for Assessment and Rating of Ataxia (SARA) scores (*r* = 0.4226, *p* = 0.0397), suggesting greater acoustic impairment with increased clinical severity.

We next examined the AVQI scores calculated from both connected speech and sustained vowel recordings. SCA patients demonstrated significantly increased AVQI scores (*p* < 0.0001) as compared to healthy controls. The control group had an average AVQI score of 2.4, while the SCA patient group showed an increased group average of 4.1, exceeding the 2.43 threshold typically associated with pathological voice quality (Figure 1A,B) [6]. Specifically, group comparisons showed significantly higher AVQI scores in all SCA subtypes relative to controls. The SCA1 group averaged 4.4, the SCA2 group 4.2, and the SCA3 group 4.0 for AVQI scores, all significantly higher than controls (*p* = 0.0482 for SCA1, *p* = 0.0112 for SCA2, and *p* = 0.0160 for SCA3; Dunn's multiple comparisons test) (Figure 1C), demonstrating that AVQI deficits occur across different SCA subtypes. Given the elevated AVQI scores observed in SCA patients, we further investigated their relationship with clinical measures of speech dysfunction using the speech item of the SARA scale. Correlation analysis revealed a significant positive association with SARA speech sub‐scores (*r* = 0.4226, *p* = 0.0397), indicating that higher AVQI scores were associated with greater clinical speech impairment (Figure 1D). These results demonstrate that AVQI scores can capture speech dysfunction across all SCA subtypes and reflect the severity of ataxic speech.

### 3.4 AVQI Sub‐Metrics

**FIGURE 2:**
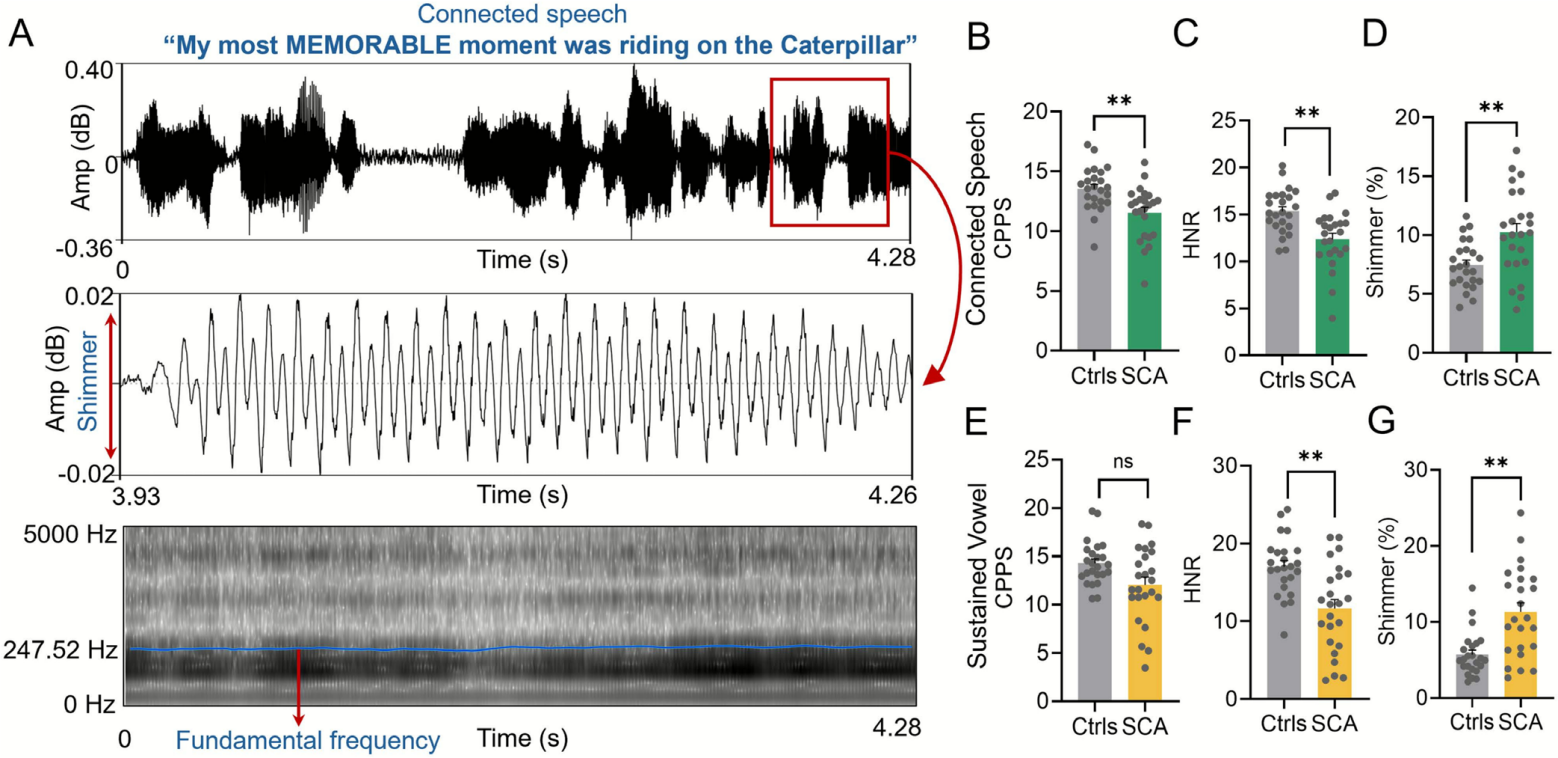
Acoustic sub‐metrics of AVQI in connected speech and sustained vowel tasks. (A) Example from a control subject showing the acoustic waveform (top), spectrogram with fundamental frequency trace (middle), and shimmer amplitude variation (bottom) during connected speech. (B–D) Connected speech sub‐metrics comparing controls and SCA patients: (B) CPPS and (C) HNR were significantly reduced, while (D) shimmer (%) was significantly increased in SCA patients. (E–G) Sustained vowel sub‐metrics: (E) CPPS showed no significant difference, (F) HNR was significantly reduced, and (G) shimmer was significantly increased in SCA patients (**p < 0.01).

Following AVQI analysis, we further examined the individual acoustic metrics that contribute to AVQI to better characterize dysfunction in both the connected speech and sustained vowel tasks (Figure 2A). Key sub‐metrics of AVQI included CPPS, HNR, and shimmer local percentage. In the connected speech task, SCA patients showed significantly reduced CPPS compared to controls (SCA: 11.53 ± 2.24; controls: 13.53 ± 1.83; *p* = 0.0040; Figure 2B). Similarly, HNR was significantly lower in SCA patients compared to controls (SCA: 12.37 ± 3.03; controls: 15.34 ± 2.33; *p* = 0.0018; Figure 2C). In contrast, shimmer values were significantly elevated in SCA patients compared to controls (SCA: 10.26% ± 3.60%; controls: 7.45% ± 2.03%; *p* = 0.0049; Figure 2D). All *p*‐values were adjusted for multiple comparisons using the Holm–Šidák correction method as described in Table S1.

For the sustained vowel task, similar changes in acoustic metrics were observed as those for connected speech (Table S2). SCA patients demonstrated a trend of decrease in CPPS, but the difference did not reach significance after the Holm–Šidák correction (*p* = 0.0507) (Figure 2E). HNR remained significantly lower in SCA patients than in controls (*p* = 0.0020) (Figure 2F), reflecting poorer harmonic structure. Finally shimmer local percentage was significantly higher in the SCA group (*p* = 0.0020) (Figure 2G), consistent with greater cycle‐to‐cycle amplitude variability. These results indicate that the sub‐metrics of AVQI are sensitive to detecting speech abnormalities across both connected speech and sustained vowel tasks, capturing distinct aspects of speech dysfunction in SCA patients.

### 3.5 Jitter

**FIGURE 3:**
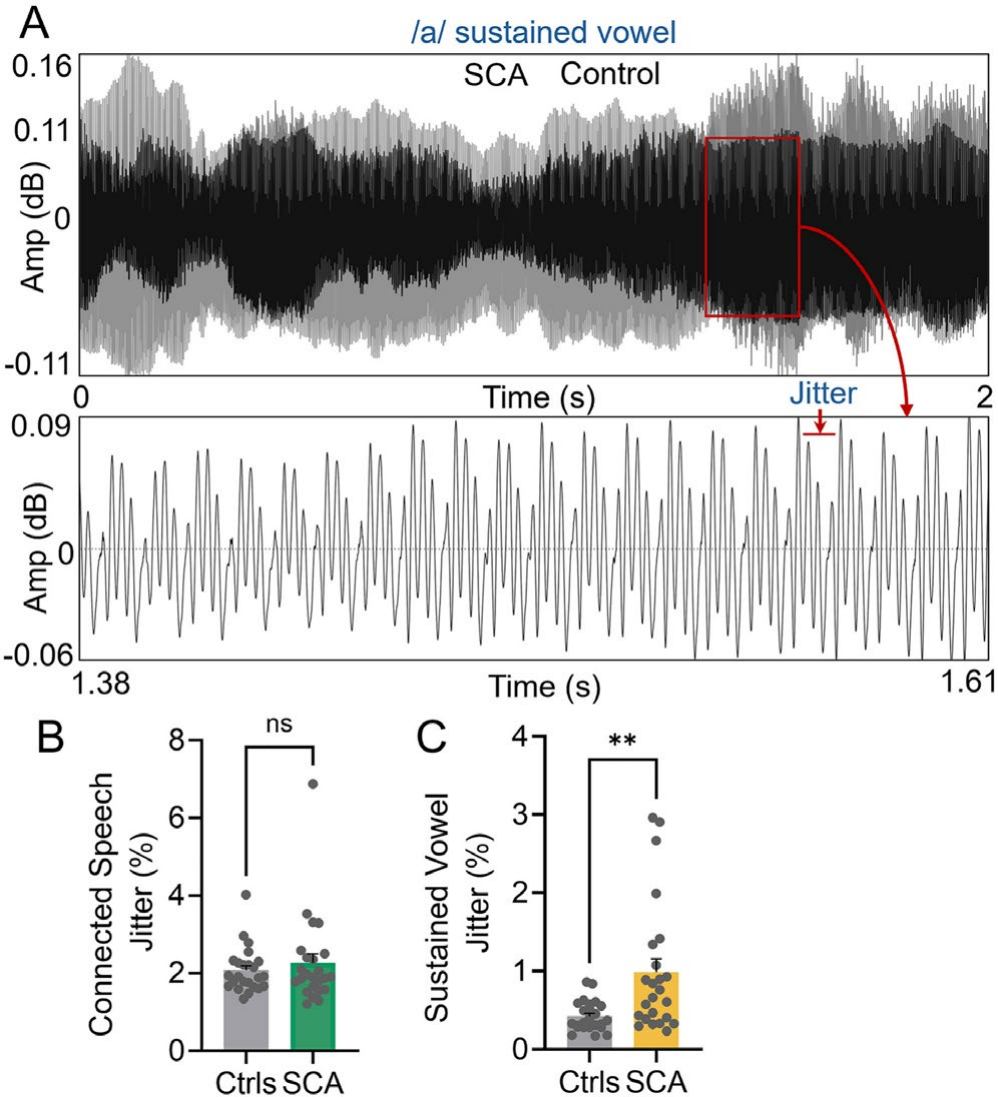
Jitter analysis in connected speech and sustained vowel tasks. (A) Overlaid waveforms of sustained vowel /a/ from a control and an SCA patient, illustrating jitter amplitude variability. The red box indicates the region shown below, highlighting cycle‐to‐cycle variations in pitch period (jitter). (B, C) Quantification of jitter (% local): (B) No significant difference was observed between controls and SCA patients during connected speech. (C) Jitter was significantly elevated in SCA patients during the sustained vowel task (**p < 0.01).

To further quantify speech dysfunction, particularly during sustained vowel production, we evaluated jitter local percentage that reflects the percentage of cycle‐to‐cycle variation in fundamental frequency. This metric was assessed across both connected speech and sustained vowel tasks (Figure 3A). During connected speech, no significant differences in jitter were observed between SCA patients and controls (Figure 3B, Table S1). However, in the sustained vowel task, jitter was significantly higher in the SCA group compared to controls, showing a 2.3‐fold increase (mean = 0.9886 for SCA vs. 0.4242 for controls; *p* = 0.0025; Figure 3C, and Table S2). These findings indicate that jitter can measure speech dysfunction during sustained vowel production but not during connected speech in SCA patients.

### 3.6 Formant Analysis

Human speech could be divided into different components using formant frequency analysis. Typically, formant frequencies (F1‐F4) range from ~300 to 5000 Hz, though values vary by vowels, anatomical differences, and age [36, 41].

**FIGURE 4:**
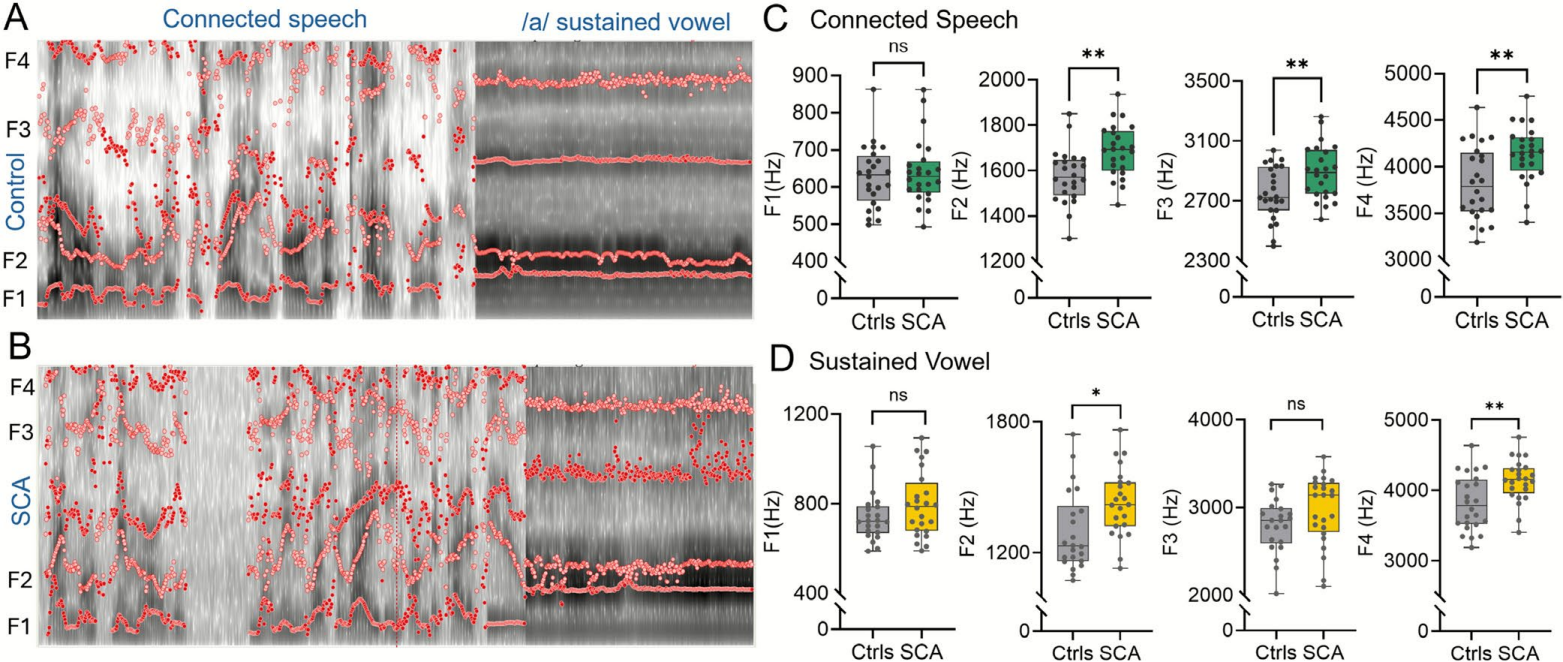
Formant frequency analysis in connected speech and sustained vowel tasks. (A, B) Representative spectrograms with overlaid formant frequency trajectories (F1–F4, red) from (A) a control and (B) an SCA patient during connected speech and sustained vowel (/a/) production. (C, D) Box plots comparing formant frequencies between controls and SCA patients for (C) connected speech and (D) sustained vowel. In connected speech, SCA patients show significantly altered F2, F3, and F4 frequencies (**p < 0.01), while in sustained vowel, differences are observed in F2 (*p < 0.05) and F4 (**p < 0.01). F1 and F3 showed no significant group differences in sustained vowel.

We next examined articulatory impairments by analyzing the first four formant frequencies (F1–F4) in both sustained vowel and connected speech tasks in SCA patients (Figure 4A–D), which reflect underlying vocal tract resonance patterns [13, 35]. Overall, SCA patients demonstrated significant shifts in formant frequencies compared to controls. In the connected speech task, F1 did not differ significantly between groups, while F2 (*p* = 0.0072), F3 (*p* = 0.0184), and F4 (*p* = 0.0087) showed significant elevation in SCA, following Holm‐Šidák correction for multiple comparisons (Figure 4C, Table S3). In the sustained vowel task, F1 and F3 showed no significant group differences, while F2 (*p* = 0.0205) and F4 (*p* = 0.0205) were significantly higher in SCA patients compared to controls, after correction for multiple comparisons (Figure 4D, Table S4), indicating robust alterations in vocal tract resonance during sustained phonation in SCA. These elevated formant frequencies in SCAs suggest dysfunctional articulatory configurations, potentially reflecting reduced control over tongue and vocal tract positioning. The observed elevations in F2–F4 in SCA patients may indicate compensatory or unstable articulatory movements affecting speech clarity in both tasks. These abnormalities likely arise from cerebellar degeneration in SCA patients, which impair the coordination and timing of speech articulation, leading to unstable vocal tract configurations and altered formant patterns.

### 3.7 Stratified Analysis According to Ataxic Speech Severity Stages

To assess if acoustic metrics capture differences in the severity of speech dysfunction, we stratified SCA patients based on their SARA speech sub‐scores into a mild group (scores 0–2; *n* = 15) and a moderate‐to‐severe group (scores 3–6; *n* = 9).

**FIGURE 5:**
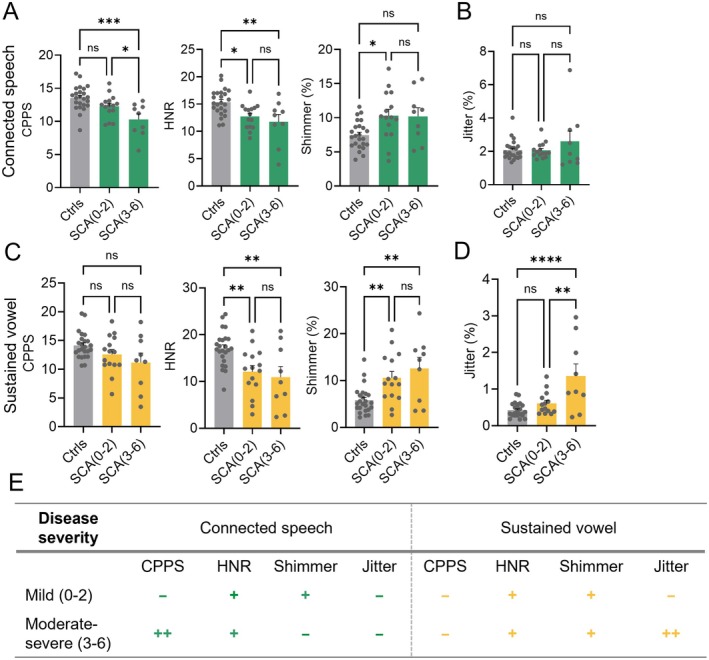
Acoustic metric stratification by SARA speech sub‐scores. Group comparisons of acoustic measures stratified by SARA speech sub‐scores: Mild (0–2) and moderate–severe (3–6). (A) For connected speech, CPPS and HNR were significantly reduced in SCA (3–6), while shimmer was significantly increased in SCA (0–2); (B) jitter showed no significant group differences. (C, D) For sustained vowel, HNR, shimmer, and jitter were significantly altered in the SCA (3–6) group compared to controls, while CPPS showed no significant differences. (E) Summary of acoustic measures highlighting differences between mild (0–2) and moderate–severe (3–6) disease severity stages ([−] no difference from controls; [+] differs from controls; [++] differs from controls and distinguishes mild vs. moderate stages) (****p < 0.0001, ***p < 0.001, **p < 0.01, *p < 0.05).

For connected speech, CPPS varied significantly by group (*F* (2, 45) = 9.27, *p* = 0.0004), with lower values in SCA (3–6) compared to both controls (*p* = 0.0003) and SCA (0–2) (*p* = 0.0499); no difference was found between SCA (0–2) and controls. HNR also showed a significant group effect (*F* (2, 45) = 7.59, *p* = 0.014), with reductions in SCA (0–2) (*p* = 0.0154) and SCA (3–6) (*p* = 0.0040) vs. controls; SCA subgroups did not differ. Shimmer showed a modest effect (*F* (2, 45) = 5.43, *p* = 0.0077), with increased values in SCA (0–2) vs. controls (*p* = 0.0145); other comparisons were not significant (Figure 5A). Jitter did not differ significantly (*F* (2, 45) = 1.30, *p* = 0.2831) (Figure 5B).

For sustained vowel, CPPS did not reach significance (*F* (2, 45) = 3.04, *p* = 0.0580), with all pairwise comparisons non‐significant. HNR showed a significant effect (*F* (2, 45) = 7.71, *p* = 0.0013), with reduced values in both SCA (0–2) (*p* = 0.0085) and SCA (3–6) (*p* = 0.0059) vs. controls; SCA subgroups did not differ. Shimmer showed a robust group effect (*F* (2, 45) = 8.99, *p* = 0.0005), with elevated values in both SCA (0–2) (*p* = 0.0088) and SCA (3–6) (*p* = 0.0015) vs. controls; no difference was found between SCA subgroups (Figure 5C). Jitter also showed a significant group effect (*F* (2, 44) = 12.93, *p* < 0.0001), with higher values in SCA (3–6) compared to both controls (*p* < 0.0001) and SCA (0–2) (*p* = 0.0017); SCA (0–2) did not differ from controls (*p* = 0.4657) (Figure 5D). Consistent with these findings, correlation analyses between these sub‐metrics, jitter, and SARA speech sub‐scores revealed similar results (Figure S2). Overall, most acoustic measures did not distinguish between mild (score 0–2) and moderate‐to‐severe (score 3–6) speech dysfunction in SCA patients. Only the CPPS, in the connected speech task, was able to capture this difference, while similarly jitter was able to track this difference in the sustained vowel task (Figure 5E).

**FIGURE 6:**
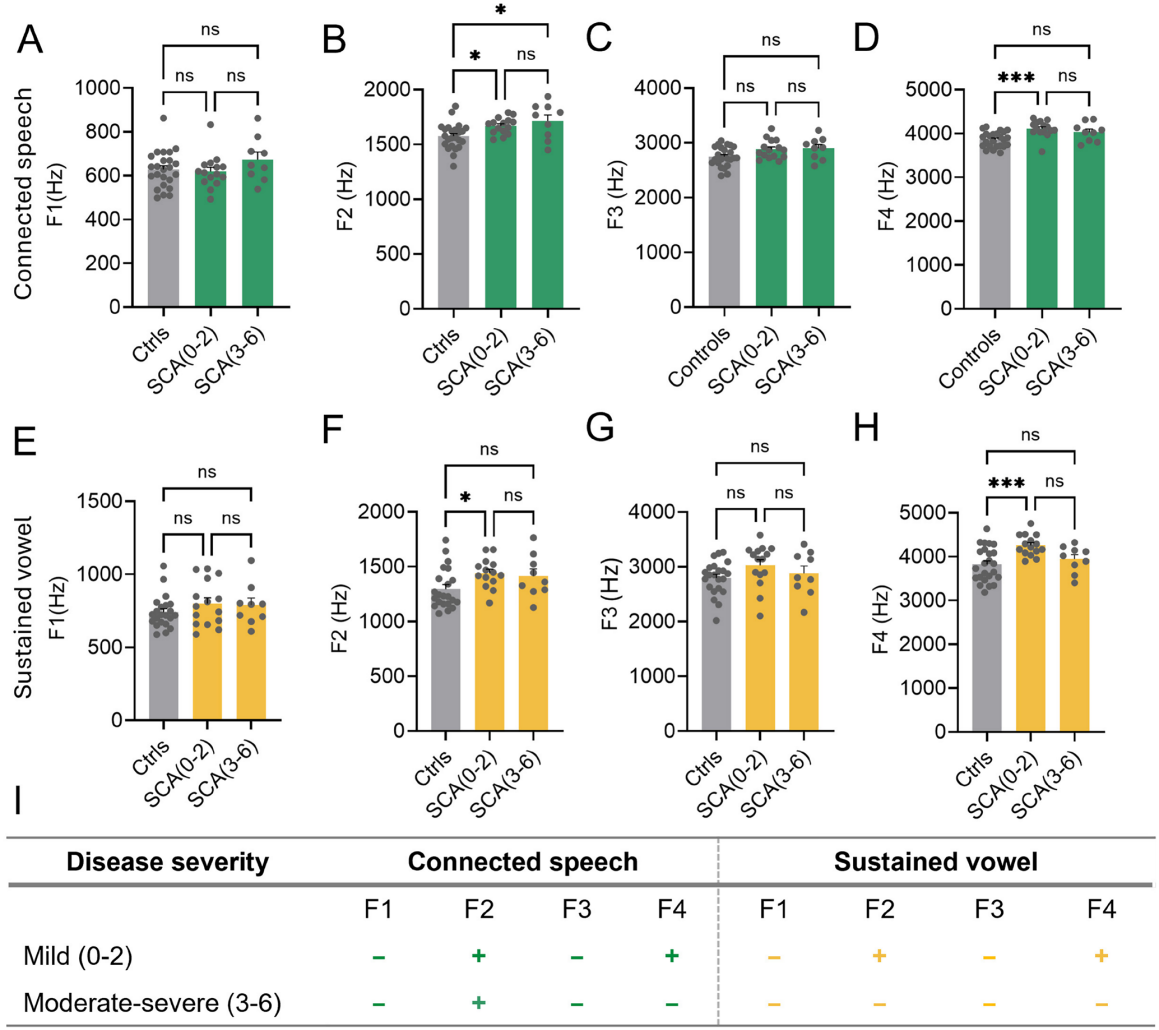
Formant frequency analysis stratified by SARA speech sub‐scores. Formant frequencies (F1–F4) were compared across controls and SCA subgroups stratified by SARA speech sub‐scores: Mild (0–2) and moderate–severe (3–6). (A–D) Connected speech: Significant reductions were observed in F2 (B, *p* < 0.05) and F4 (D, *p* < 0.001) in the SCA (0–2) group compared to controls; no significant differences were found in F1 or F3. (E–H) Sustained vowel: F2 (F, *p* < 0.05) and F4 (H, *p* < 0.001) were significantly altered in the SCA (0–2) group vs. controls, while F1 and F3 remained unchanged. (I) Summary of the formant abnormalities across both connected speech and sustained vowel tasks, highlighting differences between mild (0–2) and moderate–severe (3–6) disease severity stages ([−] no difference from controls; [+] differs from controls). No significant differences were observed between the SCA subgroups (*p < 0.05, ***p < 0.001).

Formant frequencies (F1–F4) were analyzed based on SARA speech sub‐score stratification (Figure 6). F1 (Figure 6A,E) and F3 (Figure 6C,G) showed no significant differences between SCA mild (0–2), moderate–severe (3–6) or with control groups in either connected speech or sustained vowel tasks, indicating limited value for distinguishing ataxia from controls. In connected speech, both F2 and F4 were significantly higher in mild (SCA 0–2) and moderate–severe (SCA 3–6) groups compared to controls (F2: *F* (2, 45) = 5.86, *p* = 0.0055; Tukey: SCA (0–2) vs. controls *p* = 0.0456, SCA (3–6) vs. controls *p* = 0.0106; Figure 6B; F4: *H* (2) = 14.19, *p* = 0.0008; Dunn's: SCA (0–2) vs. controls *p* = 0.0007; Figure 6D), but neither differentiated between mild and moderate–severe stages. A similar pattern was observed in the sustained vowel task, where both F2 and F4 were elevated in the mild group compared to controls (F2: Kruskal–Wallis, *H* (2) = 7.627, *p* = 0.0221; Dunn's: SCA (0–2) vs. controls *p* = 0.0251; Figure 6F; F4: *F* (2, 45) = 7.72, *p* = 0.0013; Tukey: SCA (0–2) vs. controls *p* = 0.0009; Figure 6H).

## 4 Discussion

In this study, we found that patients with SCA types 1, 2, and 3 exhibit significant speech dysfunction, with the AVQI, a multi‐parametric measure incorporating both connected speech and sustained vowels emerging as a unified and scalable marker for reflecting ataxic dysarthria across SCA subtypes.

Several studies have applied acoustic analyses to characterize speech abnormalities in patients with SCA, highlighting measurable deviations in voice quality, articulation, and prosody [5, 20, 21, 22]. Previous studies examined voice and speech characteristics in genetically confirmed SCA patients using both sustained vowel and connected speech tasks, identifying abnormalities in jitter, shimmer, and HNR, particularly in individuals with a more advanced disease state [5, 24]. In a detailed acoustic and perceptual study conducted in SCA1 patients, a significant reduced speech rate, diadochokinetic performance, as well as increased syllable duration were observed, all indicative of impaired motor timing [21]. Furthermore, in a study for SCA3 patients, monolog tasks were used to assess spontaneous speech fluency, where reduced syllable production and slowed speech were found, further supporting the utility of acoustic analysis for detecting cerebellar speech impairment [22]. While these studies highlight the value of acoustic tools in quantifying speech dysfunction in SCA, they have not comprehensively assessed the acoustic measures and identified a common acoustic metric that reliably reflects speech impairment across multiple SCA types or tracks ataxic speech severity. This study extends prior work by jointly analyzing connected speech and sustained vowels in genetically confirmed SCA1, SCA2, and SCA3, integrating AVQI and its sub‐metrics with formant‐based articulatory measures to provide a comprehensive, cross‐subtype assessment. AVQI serves as a single, scalable index that reduces task‐specific bias and enables cross‐subtype comparisons, while select sub‐metrics, such as CPPS and jitter, stratify disease severity. By identifying a unified, quantitative marker applicable across multiple SCA subtypes, our work advances the assessment of cerebellar speech dysfunction and provides a foundation for standardized monitoring and outcome measures for future clinical trials.

Our analysis of SCA1, SCA2, and SCA3 has identified a common acoustic marker of cerebellar speech dysfunction across subtypes. This approach enhances generalizability and supports standardized outcome measures for longitudinal studies and clinical trials. While it may reduce sensitivity to subtype‐specific effects, it represents a key first step toward cross‐subtype quantification. Future studies with larger cohorts and multimodal imaging–acoustic analyses will help clarify shared and distinct cerebellar circuits of speech dysfunction in SCAs.

The prolonged connected speech duration observed in SCA patients reflects disrupted motor timing, a well‐established feature of ataxic dysarthria. This aligns with earlier work documenting slowed articulation and impaired coordination in patients with various motor speech syndromes, including apraxia of speech and dysarthria associated with Parkinson's disease, Friedreich's ataxia, pure cerebellar ataxia, and multiple system atrophy [4, 42]. These highlight the cerebellum's role in the temporal organization of speech. Building on this observation, we identified AVQI as a particularly sensitive marker of speech dysfunction. AVQI scores were significantly increased in SCA subtypes 1, 2 and 3 compared to controls and exceeded the clinical threshold for pathological voice quality. This robust increase across SCA subtypes suggests that AVQI can serve as a shared indicator of disease‐related speech impairment in SCA, consistent with its use in studies associated with Parkinson's disease [6]. Sub‐metric analyses further clarified the nature of speech deficits in SCA. In both connected speech and sustained vowel tasks, SCA patients exhibited significantly reduced CPPS and HNR values, indicating diminished harmonic clarity and increased breathiness, key features of ataxic dysarthria [43]. Shimmer values were consistently elevated across tasks, reflecting greater amplitude instability. Interestingly, jitter, a measure of cycle‐to‐cycle frequency variation, was unaltered during connected speech yet increased during sustained vowel phonation, suggesting that steady‐state speech tasks may be more sensitive to capturing subtle laryngeal deficits. This task‐specific sensitivity of jitter aligns with prior studies highlighting differences in speech control demands between connected and sustained speech in ataxic dysarthria [4]. This pattern suggests that task selection may modulate the sensitivity of specific acoustic markers and supports incorporating both task types in comprehensive speech evaluation protocols. These results suggest that while CPPS, HNR, and shimmer reflect general speech dysfunction, jitter may be particularly useful for assessing sustained vowel phonation deficits in SCA.

Additionally, as this study did not directly assess physiological mechanisms, the observed phonatory changes (elevated AVQI with reduced CPPS/HNR and increased shimmer across tasks, and jitter elevations most evident during sustained vowels) may support the hypothesis that unstable subglottal pressure from neuro‐respiratory involvement [44, 45], together with cerebellar discoordination, drives breathy/rough phonation and amplitude instability, while sustained phonation unmasks jitter in SCA. Future studies combining acoustic, respiratory, and imaging measures could clarify these mechanistic links.

Formant frequency analysis provided critical insight into the articulatory disturbances underlying the speech dysfunctions. Formants reflect resonant frequencies shaped by the configuration and movement of the vocal tract, and their deviations can serve as indirect markers of articulatory control. In our study, SCA patients showed elevated F2–F4 formant frequencies for the tasks, indicating impaired coordination of tongue advancement, lip and jaw positioning, and fine articulatory movements. Previous research found F1–F2 frequencies demonstrating dysfunctional sound vowel articulation in SCA [46]. Automated formant tracking in connected speech of cerebellar ataxia patients also revealed consistent vowel articulation abnormalities, highlighting the value of formant‐based analysis [35] Our findings extend this literature by demonstrating that similar formant deviations occur across multiple genetically confirmed SCA subtypes, in both connected speech and sustained vowel tasks, including higher formants (F3–F4) that reflect finer articulatory control. This establishes a shared articulatory dysfunction pattern linked to cerebellar degeneration, reflecting the cerebellum's role in fine motor control of speech [3, 47] and complements voice quality metrics like AVQI by providing insights into the spatial and temporal aspects of articulation in SCA.

Furthermore, AVQI scores and speech duration in SCA patients both correlate significantly with SARA speech sub‐scores, demonstrating their potential as markers of speech impairment severity. We further investigated which acoustic measures can track speech dysfunction severity and identified sub‐metric‐specific differences across disease stages. In the connected speech task, CPPS, a sub‐metric of AVQI was able to differentiate between severity levels, while in the sustained vowel task, jitter showed a similar ability to track speech dysfunction. However, other sub‐metrics and formant frequencies (F1–F4) could not distinguish between different severity stages. Further studies are needed to determine which acoustic measures can reliably track disease progression over time. Additionally, as controls were age‐matched, confounding from age‐related voice changes was minimized; nonetheless, older SCA cohorts may show slightly higher AVQI and lower CPPS than observed here. Future age‐stratified analyses may help distinguish normative aging from disease‐related effects.

Our study has several limitations. First, the sample size for each SCA subtype was modest and unevenly distributed, which may have limited our ability to detect subtle subtype‐specific differences. In addition, future studies with larger cohorts should also examine gender effects on acoustic measures to validate and extend our findings. Second, subsequent studies could incorporate additional speech intelligibility ratings or patient‐reported outcomes such as the Voice Handicap Index. In addition, assessing how acoustic measures relate to specific categories of the CCAS could offer additional complementary clinical insights. Third, the speech samples used focused on a limited set of tasks. Future research should investigate the robustness of these acoustic markers across various speech tasks, such as monologs, repeated word tasks, or alternative sustained vowel productions. Fourth, the increased speech duration may result from multiple factors, including impaired timing, rhythm, voicing, or pausing, which our current analysis could not disentangle. Timing‐ and coordination‐based features, such as diadochokinetic rates or voice‐onset timing, were beyond the scope of this study, as our primary aim was to focus on phonatory and articulatory measures, to establish objective, cross‐subtype markers of speech impairment. Future studies incorporating timing‐based analyses will be valuable for elucidating how cerebellar degeneration disrupts the temporal organization of speech. Finally, longitudinal studies would be valuable in tracking the progression of speech dysfunction over time, potentially offering insights into information for disease dynamics and early intervention.

## 5 Conclusion

This study demonstrates that AVQI is a common acoustic marker, for detecting and measuring speech dysfunction objectively in SCA patients across subtypes 1, 2, and 3. In addition, acoustic sub‐metrics of AVQI (CPPS, HNR, and shimmer), along with jitter and formant frequencies, effectively captured different aspects of speech and articulatory dysfunction. Jitter during sustained vowel tasks reflected phonatory instability, while formant frequency deviations highlighted articulatory impairments. Further, the CPPS sub‐metric and jitter are among the only acoustic measures that can capture differences in the severity of speech dysfunction. Together, these measures offer insights into speech dysfunction in SCAs and the use of acoustic tools for objective, comprehensive speech assessment, with potential use in disease monitoring to guide targeted therapeutic interventions.

## Author Contributions

Conceptualization: Z.F., S.‐H.K., and A.K. Methodology: Z.F., C.H., S.‐H.K., and A.K. Investigation: Z.F., C.H., S.‐H.K., and A.K. Visualization: Z.F., C.H., S.‐H.K., and A.K. Funding acquisition: S.‐H.K. Project administration: Z.F., C.H., H.L., P.P., and A.K. Supervision: S.‐H.K. and A.K. Writing‐original draft: Z.F., C.H., and A.K. Writing‐review and editing: Z.F., C.H., H.L., P.P., S.‐H.K., and A.K.

## Funding

This study was supported by the International Essential Tremor Foundation and National Institute of Neurological Disorders and Stroke (R01 NS124854, R01 NS136686, and UE5 NS070697).

## Conflicts of Interest

The authors declare no conflicts of interest.

## Supporting information


**Figure S1:** Duration of connected speech.

**Figure S2:** Correlation of sub‐metrics and jitter with SARA speech sub‐scores in SCA patients.

**Table S1:** Multiple comparison correction for sub‐metrics of AVQI and Jitter (connected speech).
**Table S2:** Multiple comparison correction for sub‐metrics of AVQI and Jitter (sustained vowel).
**Table S3:** Multiple comparison correction for formants (connected speech).
**Table S4:** Multiple comparison correction for formants (sustained vowel).

## Data Availability

The data that support the findings of this study are available from the corresponding author upon reasonable request.

## References

[1] S. Radmard , T. A. Zesiewicz , and S. H. Kuo , “Evaluation of Cerebellar Ataxic Patients,” Neurologic Clinics 41, no. 1 (2023): 21–44, 10.1016/j.ncl.2022.05.002. 36400556 PMC10354692

[2] C. Y. R. Lin and S. H. Kuo , “Ataxias: Hereditary, Acquired, and Reversible Etiologies,” Seminars in Neurology 43, no. 1 (2023): 48–64, 10.1055/s-0043-1763511. 36828010 PMC10354687

[3] H. Ackermann , K. Mathiak , and A. Riecker , “The Contribution of the Cerebellum to Speech Production and Speech Perception: Clinical and Functional Imaging Data,” Cerebellum 6, no. 3 (2007): 202–213, 10.1080/14734220701266742. 17786816

[4] R. D. Kent , J. F. Kent , J. R. Duffy , J. E. Thomas , G. Weismer , and S. Stuntebeck , “Ataxic Dysarthria,” Journal of Speech, Language, and Hearing Research 43, no. 5 (2000): 1275–1289, 10.1044/jslhr.4305.1275. 11063247

[5] E. Schalling and L. Hartelius , “Speech in Spinocerebellar Ataxia,” Brain and Language 127, no. 3 (2013): 317–322, 10.1016/j.bandl.2013.10.002. 24182841

[6] Y. Maryn and D. Weenink , “Objective Dysphonia Measures in the Program Praat: Smoothed Cepstral Peak Prominence and Acoustic Voice Quality Index,” Journal of Voice 29, no. 1 (2015): 35–43, 10.1016/j.jvoice.2014.06.015. 25499526

[7] Y. Maryn , P. Corthals , P. Van Cauwenberge , N. Roy , and M. De Bodt , “Toward Improved Ecological Validity in the Acoustic Measurement of Overall Voice Quality: Combining Continuous Speech and Sustained Vowels,” Journal of Voice 24, no. 5 (2010): 540–555, 10.1016/j.jvoice.2008.12.014. 19883993

[8] Y. Maryn , N. Roy , M. De Bodt , P. Van Cauwenberge , and P. Corthals , “Acoustic Measurement of Overall Voice Quality: A Meta‐Analysis,” Journal of the Acoustical Society of America 126, no. 5 (2009): 2619–2634, 10.1121/1.3224706. 19894840

[9] Y. Maryn , M. De Bodt , and N. Roy , “The Acoustic Voice Quality Index: Toward Improved Treatment Outcomes Assessment in Voice Disorders,” Journal of Communication Disorders 43, no. 3 (2010): 161–174, 10.1016/j.jcomdis.2009.12.004. 20080243

[10] Y. D. Heman‐Ackah , D. D. Michael , and G. S. J. Goding , “The Relationship Between Cepstral Peak Prominence and Selected Parameters of Dysphonia,” Journal of Voice 16, no. 1 (2002): 20–27, 10.1016/s0892-1997(02)00067-x. 12008652

[11] E. Yumoto , W. J. Gould , and T. Baer , “Harmonics‐to‐Noise Ratio as an Index of the Degree of Hoarseness,” Journal of the Acoustical Society of America 71, no. 6 (1982): 1544–1549, 10.1121/1.387808. 7108029

[12] J. P. Teixeira , C. Oliveira , and C. Lopes , “Vocal Acoustic Analysis—Jitter, Shimmer and HNR Parameters,” Procedia Technology 9 (2013): 1112–1122, 10.1016/j.protcy.2013.12.124.

[13] Y. Yunusova , G. Weismer , R. D. Kent , and N. M. Rusche , “Breath‐Group Intelligibility in Dysarthria: Characteristics and Underlying Correlates,” Journal of Speech, Language, and Hearing Research 48, no. 6 (2005): 1294–1310, 10.1044/1092-4388(2005/090). 16478372

[14] M. Farrús and J. Hernando , “Using Jitter and Shimmer in Speaker Verification,” IET Signal Processing 3, no. 4 (2009): 247–257, 10.1049/iet-spr.2008.0147.

[15] Y. Yunusova , J. R. Green , L. Greenwood , J. Wang , G. L. Pattee , and L. Zinman , “Tongue Movements and Their Acoustic Consequences in Amyotrophic Lateral Sclerosis,” Folia Phoniatrica et Logopaedica 64, no. 2 (2012): 94–102, 10.1159/000336890. 22555651 PMC3369262

[16] M. F. Maffei , J. R. Green , O. Murton , et al., “Acoustic Measures of Dysphonia in Amyotrophic Lateral Sclerosis,” Journal of Speech, Language, and Hearing Research 66, no. 3 (2023): 872–887, 10.1044/2022_JSLHR-22-00363. PMC10205101 36802910

[17] A. M. Goberman and C. Coelho , “Acoustic Analysis of Parkinsonian Speech I: Speech Characteristics and L‐Dopa Therapy,” NeuroRehabilitation 17, no. 3 (2002): 237–246. 12237505

[18] R. B. Convey , A. M. Laukkanen , S. Ylinen , and N. Penttilä , “Analysis of Voice in Parkinson's Disease Utilizing the Acoustic Voice Quality Index,” Journal of Voice (forthcoming), 10.1016/j.jvoice.2023.12.025. 38242818

[19] G. Moya‐Galé , J. Spielman , L. A. Ramig , L. Campanelli , and Y. Maryn , “The Acoustic Voice Quality Index (AVQI) in People With Parkinson's Disease Before and After Intensive Voice and Articulation Therapies: Secondary Outcome of a Randomized Controlled Trial,” Journal of Voice 38, no. 6 (2024): 1529.e7–e16, 10.1016/j.jvoice.2022.03.014. PMC9576821 35450735

[20] G. Di Rauso , A. Castellucci , F. Cavallieri , et al., “Speech, Gait, and Vestibular Function in Cerebellar Ataxia With Neuropathy and Vestibular Areflexia Syndrome,” Brain Sciences 13, no. 10 (2023): 1467, 10.3390/brainsci13101467. 37891834 PMC10605709

[21] T. van Prooije , S. Knuijt , J. Oostveen , K. Kapteijns , A. P. Vogel , and B. van de Warrenburg , “Perceptual and Acoustic Analysis of Speech in Spinocerebellar Ataxia Type 1,” Cerebellum 23, no. 1 (2024): 112–120, 10.1007/s12311-023-01513-9. 36633828 PMC10864471

[22] V. B. Dos Santos , A. Ayres , M. L. M. Kieling , et al., “Differences in Spontaneous Speech Fluency Between Parkinson's Disease and Spinocerebellar Ataxia Type 3,” Frontiers in Neurology 14 (2023): 1179287, 10.3389/fneur.2023.1179287. 37213898 PMC10196352

[23] E. Schalling and L. Hartelius , “Acoustic Analysis of Speech Tasks Performed by Three Individuals With Spinocerebellar Ataxia,” Folia Phoniatrica et Logopaedica 56, no. 6 (2004): 367–380, 10.1159/000081084. 15557775

[24] E. Schalling , B. Hammarberg , and L. Hartelius , “Perceptual and Acoustic Analysis of Speech in Individuals With Spinocerebellar Ataxia (SCA),” Logopedics, Phoniatrics, Vocology 32, no. 1 (2007): 31–46, 10.1080/14015430600789203. 17454658

[25] H. T. Orr , M. Y. Chung , S. Banfi , et al., “Expansion of an Unstable Trinucleotide CAG Repeat in Spinocerebellar Ataxia Type 1,” Nature Genetics 4, no. 3 (1993): 221–226, 10.1038/ng0793-221. 8358429

[26] D. Lorenzetti , S. Bohlega , and H. Y. Zoghbi , “The Expansion of the CAG Repeat in Ataxin‐2 Is a Frequent Cause of Autosomal Dominant Spinocerebellar Ataxia,” Neurology 49, no. 4 (1997): 1009–1013, 10.1212/wnl.49.4.1009. 9339681

[27] A. M. Sidky , A. R. V. Melo , T. T. Kay , M. Raposo , M. Lima , and D. G. Monckton , “Age‐Dependent Somatic Expansion of the ATXN3 CAG Repeat in the Blood and Buccal Swab DNA of Individuals With Spinocerebellar Ataxia Type 3/Machado‐Joseph Disease,” Human Genetics 143, no. 11 (2024): 1363–1378, 10.1007/s00439-024-02698-7. 39375222 PMC11522074

[28] T. Schmitz‐Hübsch , S. T. du Montcel , L. Baliko , et al., “Scale for the Assessment and Rating of Ataxia: Development of a New Clinical Scale,” Neurology 66, no. 11 (2006): 1717–1720, 10.1212/01.wnl.0000219042.60538.92. 16769946

[29] P. Boersma and V. van Heuven , “Speak and unSpeak With Praat,” Glot International 5, no. 9–10 (2001): 341–347.

[30] B. van der Woerd , M. Wu , V. Parsa , P. C. Doyle , and K. Fung , “Evaluation of Acoustic Analyses of Voice in Nonoptimized Conditions,” Journal of Speech, Language, and Hearing Research 63, no. 12 (2020): 3991–3999, 10.1044/2020_JSLHR-20-00212. 33186510

[31] A. Portnova , A. Fletcher , A. Wisler , and S. A. Borrie , “Assessing Fundamental Frequency Variation in Speakers With Parkinson's Disease: Effects of Tracking Errors,” Journal of Speech, Language, and Hearing Research 68 (2025): 3568–3582, 10.1044/2024_JSLHR-24-00381. PMC12337113 39970199

[32] G. H. Kim , D. W. Lim , J. W. Kim , H. J. Park , and Y. W. Lee , “A Cepstral Analysis of Pathological Voice Quality in the Korean Population Using Praat,” Journal of Voice 39, no. 2 (2025): 559.e19–e27, 10.1016/j.jvoice.2022.10.011. 36464574

[33] R. Patel , K. Connaghan , D. Franco , et al., “‘The Caterpillar’: A Novel Reading Passage for Assessment of Motor Speech Disorders,” American Journal of Speech‐Language Pathology 22, no. 1 (2013): 1–9, 10.1044/1058-0360(2012/11-0134). 22846881

[34] Y. Kim , H. Chung , and A. Thompson , “Acoustic and Articulatory Characteristics of English Semivowels /ɹ, l, w/ Produced by Adult Second‐Language Speakers,” Journal of Speech, Language, and Hearing Research 65, no. 3 (2022): 890–905, 10.1044/2021_JSLHR-21-00152. 35104414

[35] V. Illner , T. Tykalova , D. Skrabal , J. Klempir , and J. Rusz , “Automated Vowel Articulation Analysis in Connected Speech Among Progressive Neurological Diseases, Dysarthria Types, and Dysarthria Severities,” Journal of Speech, Language, and Hearing Research 66, no. 8 (2023): 2600–2621, 10.1044/2023_JSLHR-22-00526. 37499137

[36] P. Ladefoged and K. Johnson , A Course in Phonetics, Seventh ed. (Cengage Learning Inc, 2014).

[37] K. N. Stevens , Acoustic Phonetics, vol. 30 (MIT Press, 2000).

[38] R. D. Kent and H. K. Vorperian , “Static Measurements of Vowel Formant Frequencies and Bandwidths: A Review,” Journal of Communication Disorders 74 (2018): 74–97, 10.1016/j.jcomdis.2018.05.004. 29891085 PMC6002811

[39] C. Dromey , G. O. Jang , and K. Hollis , “Assessing Correlations Between Lingual Movements and Formants,” Speech Communication 55, no. 2 (2013): 315–328, 10.1016/j.specom.2012.09.001.

[40] A. S. Mefferd , “Associations Between Tongue Movement Pattern Consistency and Formant Movement Pattern Consistency in Response to Speech Behavioral Modifications,” Journal of the Acoustical Society of America 140, no. 5 (2016): 3728–3737, 10.1121/1.4967446. 27908069 PMC5392073

[41] R. D. Kent and C. Read , *The Acoustic Analysis of Speech* , Second ed. (Delmar Cengage Learning, 2002).

[42] W. Ziegler , “Task‐Related Factors in Oral Motor Control: Speech and Oral Diadochokinesis in Dysarthria and Apraxia of Speech,” Brain and Language 80, no. 3 (2002): 556–575, 10.1006/brln.2001.2614. 11896657

[43] B. E. Murdoch , D. G. Theodoros , P. D. Stokes , and H. J. Chenery , “Abnormal Patterns of Speech Breathing in Dysarthric Speakers Following Severe Closed Head Injury,” Brain Injury 7, no. 4 (1993): 295–308, 10.3109/02699059309034956. 8358403

[44] D. D. Biswas , L. El Haddad , R. Sethi , et al., “Neuro‐Respiratory Pathology in Spinocerebellar Ataxia,” Journal of the Neurological Sciences 443 (2022): 120493, 10.1016/j.jns.2022.120493. 36410186 PMC9808489

[45] C. F. Viana , C. S. Jaques , M. L. Escorcio‐Bezerra , J. L. Pedroso , and O. G. P. Barsottini , “Respiratory Evaluation in Spinocerebellar Ataxia Type 2,” Cerebellum 24, no. 4 (2025): 98, 10.1007/s12311-025-01845-8. 40358860

[46] S. Skodda , “Vowel Articulation in Patients With Spinocerebellar Ataxia,” International Journal of Speech and Language Pathology and Audiology 1 (2014): 63–71, 10.12970/2311-1917.2013.01.02.3.

[47] K. A. Spencer and D. L. Slocomb , “The Neural Basis of Ataxic Dysarthria,” Cerebellum 6, no. 1 (2007): 58–65, 10.1080/14734220601145459. 17366266

