# Supplementary figures and images for: Acoustic Measures Capture Speech Dysfunction in Spinocerebellar Ataxia
Source: Ann Clin Transl Neurol. 2025 Nov 28;13(4):807–18. doi: 10.1002/acn3.70264 (PMC13071143; doi:10.1002/acn3.70264)

A

My most MEMORABLE moment was riding on the Caterpillar

Connected speech (Sec)

B

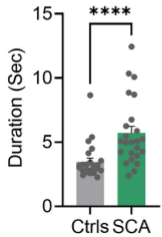

C

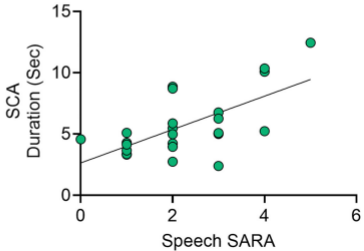

Supplement: Supplementary file 1 — Figure S1: Duration of connected speech. [file ACN3-13-807-s002.pdf]

**A**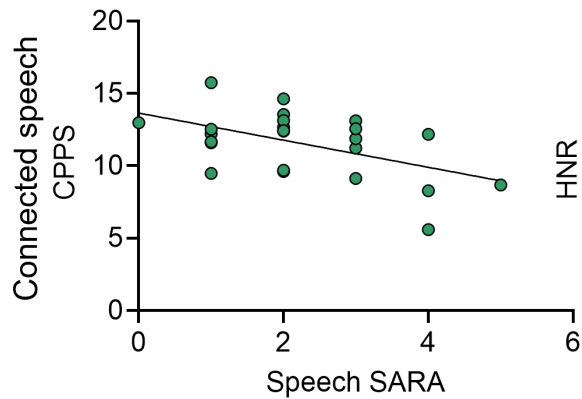**B**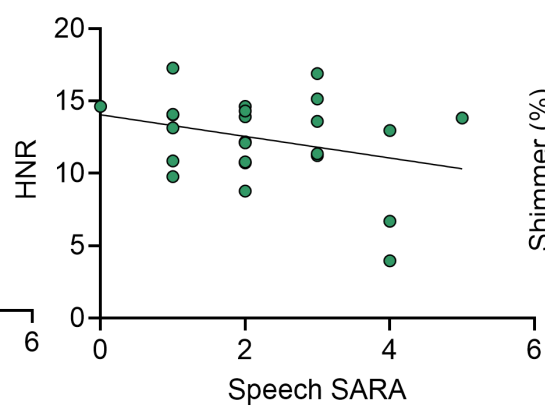**C**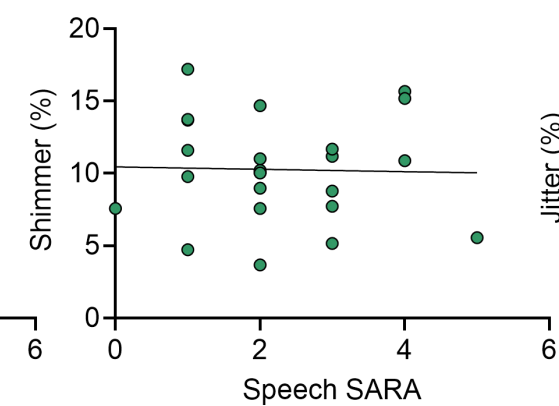**D**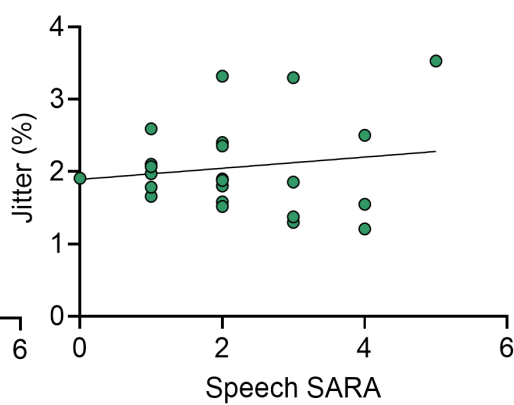**E**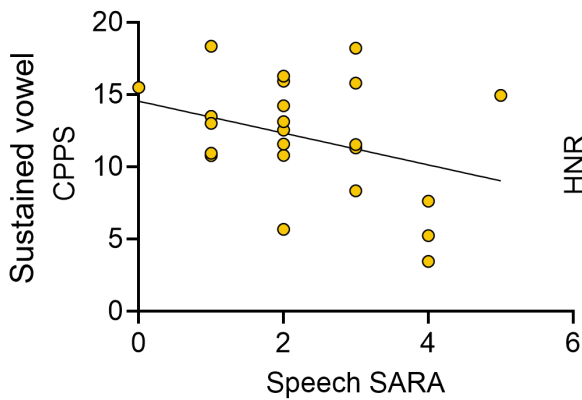**F**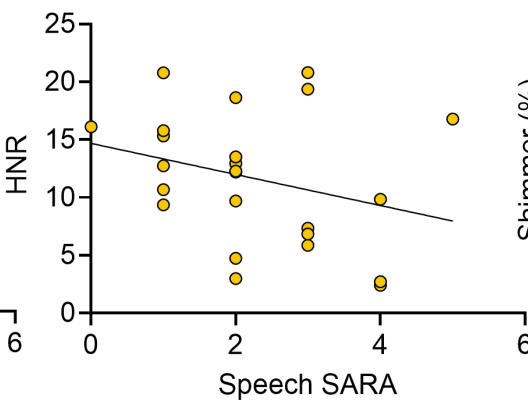**G**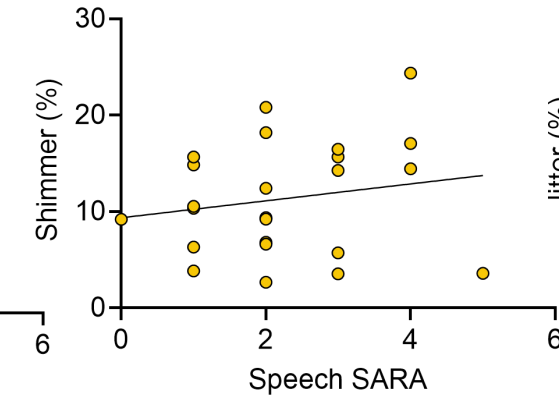**H**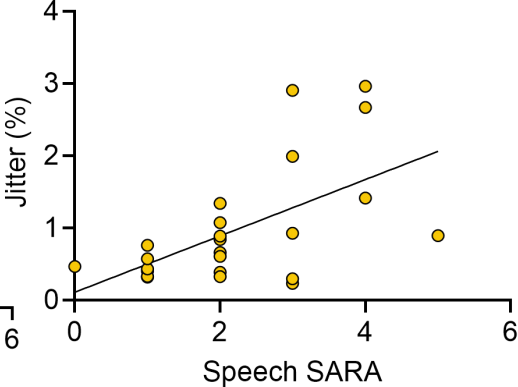

Supplement: Supplementary file 2 — Figure S2: Correlation of sub‐metrics and jitter with SARA speech sub‐scores in SCA patients. [file ACN3-13-807-s001.pdf]
