# Supplementary material for: Acoustic Measures Capture Speech Dysfunction in Spinocerebellar Ataxia
Source: Ann Clin Transl Neurol. 2025 Nov 28;13(4):807–18. doi: 10.1002/acn3.70264 (PMC13071143; doi:10.1002/acn3.70264)
Supplement: Supplementary file 3 — Table S1: Multiple comparison correction for sub‐metrics of AVQI and Jitter (connected speech). Table S2: Multiple comparison correction for sub‐metrics of AVQI and Jitter (sustained vowel). Table S3: Multiple comparison correction for formants (connected speech). Table S4: Multiple comparison correction for formants (sustained vowel). [file ACN3-13-807-s003.docx]

**Supplementary Information**

**Supplementary Table 1: Multiple comparison correction for sub-metrics of AVQI and Jitter (connected speech)**

| Measure | Mean rank (controls) | Mean rank (SCA) | Mean rank difference | Mann-Whitney U | *p*-value | Adjusted  *p-*value | Significant? |
| --- | --- | --- | --- | --- | --- | --- | --- |
| CPPS | 30.83 | 18.17 | 12.67 | 136.0 | 0.0013 | 0.0040 | Yes |
| HNR | 31.38 | 17.63 | 13.75 | 123.0 | 0.00045 | 0.0018 | Yes |
| Shimmer | 18.50 | 30.50 | -12.00 | 144.0 | 0.0024 | 0.0049 | Yes |
| Jitter | 24.50 | 24.50 | 0.000 | 288.0 | >0.9999 | >0.9999 | No |

Statistical summary of Mann-Whitney U-tests comparing controls and SCA patients in connected speech measures, with Holm-Šídák multiple comparison method.

**Supplementary Table 2: Multiple comparison correction for sub-metrics of AVQI and Jitter (sustained vowel)**

| Measure | Mean rank (controls) | Mean rank (SCA) | Mean rank difference | Mann-Whitney U | *p*-value | Adjusted  *p-*value | Significant? |
| --- | --- | --- | --- | --- | --- | --- | --- |
| CPPS | 28.46 | 20.54 | 7.917 | 193.0 | 0.0507 | 0.0507 | No |
| HNR | 31.21 | 17.79 | 13.42 | 127.0 | 0.0006 | 0.0020 | Yes |
| Shimmer | 17.67 | 31.33 | -13.67 | 124.0 | 0.0005 | 0.0020 | Yes |
| Jitter | 18.13 | 30.88 | -12.75 | 135.0 | 0.0012 | 0.0025 | Yes |

Statistical summary of Mann-Whitney U-tests comparing controls vs SCA patients in sustained vowel measures, with Holm-Šídák multiple comparison method.

**Supplementary Table 3: Multiple comparison correction for formants (connected speech)**

| Formants | Mean controls | Mean SCA | Difference | SE of difference | t ratio | *p-*value | Adjusted *p*-value | Significant? |
| --- | --- | --- | --- | --- | --- | --- | --- | --- |
| F1 | 628.2 | 639.7 | -11.51 | 24.91 | 0.4619 | 0.6463 | 0.6463 | No |
| F2 | 1575 | 1687 | -111.5 | 33.71 | 3.309 | 0.0018 | 0.0072 | Yes |
| F3 | 2749 | 2889 | -140.2 | 51.64 | 2.715 | 0.0092 | 0.0184 | Yes |
| F4 | 3821 | 4140 | -318.6 | 101.4 | 3.142 | 0.0029 | 0.0087 | Yes |

Statistical summary of Unpaired t-tests comparing controls vs SCA patients in connected speech measures, with Holm-Šídák multiple comparison method.

**Supplementary Table 4: Multiple comparison correction for formants (sustained vowel)**

| Formants | Mean rank (controls) | Mean rank (SCA) | Mean rank difference | Mann-Whitney U | *p*-value | Adjusted  *p*-value | Significant? |
| --- | --- | --- | --- | --- | --- | --- | --- |
| F1 | 20.86 | 25.92 | -5.053 | 206 | 0.2082 | 0.2082 | No |
| F2 | 17.59 | 28.17 | -10.58 | 134 | 0.0062 | 0.0205 | Yes |
| F3 | 19.68 | 27 | -7.318 | 180 | 0.0659 | 0.1276 | No |
| F4 | 18.92 | 30.08 | -11.17 | 154 | 0.0051 | 0.0205 | Yes |

Statistical summary of Mann-Whitney U-tests comparing controls vs SCA patients in sustained vowel measures, with Holm-Šídák multiple comparison method.

.
